# Enantioselective Biosynthesis of L-Phenyllactic Acid From Phenylpyruvic Acid *In Vitro* by L-Lactate Dehydrogenase Coupling With Glucose Dehydrogenase

**DOI:** 10.3389/fbioe.2022.846489

**Published:** 2022-02-18

**Authors:** Dong Zhang, Ting Zhang, Yuqing Lei, Wenqian Lin, Xingyi Chen, Minchen Wu

**Affiliations:** ^1^ Key Laboratory of Carbohydrate Chemistry and Biotechnology, Ministry of Education, School of Biotechnology, Jiangnan University, Wuxi, China; ^2^ Haiyan Food and Drug Inspection and Testing Center, Haiyan, China; ^3^ Wuxi School of Medicine, Jiangnan University, Wuxi, China

**Keywords:** L-PLA, coenzyme regeneration system, L-lactate dehydrogenase, fed-batch, asymmetric reduction

## Abstract

As a valuable versatile building block, L-phenyllactic acid (L-PLA) has numerous applications in the fields of agriculture, pharmaceuticals, and biodegradable plastics. However, both normally chemically synthesized and naturally occurring PLA are racemic, and the production titer of L-PLA is not satisfactory. To improve L-PLA production and reduce the high cost of NADH, an *in vitro* coenzyme regeneration system of NADH was achieved using the glucose dehydrogenase variant *Ls*GDH^D255C^ and introduced into the L-PLA production process. Here an NADH-dependent L-lactate dehydrogenase-encoding variant gene (L-*Lcldh*1^Q88A/I229A^) was expressed in *Pichia pastoris* GS115. The specific activity of L-*Lc*LDH1^Q88A/I229A^ (Pp) was as high as 447.6 U/mg at the optimum temperature and pH of 40°C and 5.0, which was 38.26-fold higher than that of wild-type L-*Lc*LDH1 (Pp). The catalytic efficiency (*k*_cat_/*K*_m_) of L-*Lc*LDH1^Q88A/I229A^ (Pp) was 94.3 mM^−1^ s^−1^, which was 67.4- and 25.5-fold higher than that of L-*Lc*LDH1(Pp) and L-*Lc*LDH1^Q88A/I229A^ (Ec) expressed in *Escherichia coli*, respectively*.* Optimum reactions of L-PLA production by dual-enzyme catalysis were at 40°C and pH 5.0 with 10.0 U/ml L-*Lc*LDH1^Q88A/I229A^ (Pp) and 4.0 U/ml *Ls*GDH^D255C^. Using 0.1 mM NAD^+^, 400 mM (65.66 g/L) phenylpyruvic acid was completely hydrolyzed by fed-batch process within 6 h, affording L-PLA with 90.0% yield and over 99.9% *ee*_p_. This work would be a promising technical strategy for the preparation of L-PLA at an industrial scale.

## Introduction

Phenyllactic acid (PLA), a natural organic acid with high value added, is considered a promising preservative widely used in food and feed due to its broad antimicrobial activity (Ning et al., 2017). The optically pure PLA is also a high-value-added chiral compound with potential applications in the pharmaceutical and biomaterial areas—for example, L-PLA was applied to synthesize non-amino acid statine, protease inhibitors, and anti-HIV reagents (Kano et al., 1988; Urban and Moore, 1992; Fujita et al., 2013), while D-PLA was also applied as a building block in biopolyester polyhydroxyalkanoate and a hypoglycemic agent englitazone (Xu et al., 2016; Yang et al., 2018). Additionally, L-PLA is a promising building block for bio-based materials, as L-PLA can be polymerized into the biopolymer poly(L-phenyllactic acid)s. Unlike poly(L-lactic acid)s, poly(L-phenyllactic acid)s exhibit high-ultraviolet-absorbing properties due to the bulky aromatic side chain at the C-3 group of L-lactic acid, which has been applied in plastics, pharmaceutics, and agrochemistry in terms of its superior chemical–physical and antimicrobial properties (Chifiriuc et al., 2007; Zheng et al., 2015). Due to its potential application for the synthesis of aromatic polymers, L-PLA production has aroused great interest recently.

With the increasing environmental awareness, biocatalysis using enzymes or whole cells has attracted much attention, given its properties such as high enantioselectivity (*ee*_p_ >99%), 100% theoretical yield, no or little byproducts, and being an environment-friendly process (Hou et al., 2019). To date, various biotechnological methods have been developed for L-PLA production, mainly including microbial fermentation and enzymatic/whole-cell cascade biocatalysis (Zheng et al., 2013). In general, L-PLA is mainly produced by L-lactate dehydrogenases (LDHs), owing to the above-mentioned advantages. However, few L-LDHs exhibited a high catalytic activity, catalytic efficiency (*k*_cat_/*K*_m_), and yield, especially towards the bulkier substrates such as phenylpyruvic acid (PPA), which made them unable to be utilized effectively—for instance, the activities of L-*Lp*LDH from *Lactobacillus plantarum* and L-*Bs*LDH from *Geobacillus stearothermophilus* were 28.11 and 7.39 U/mg wet cell, respectively (Aslan et al., 2016; Zhu et al., 2017). In addition, by fed-batch conversion, the highest PLA production reached 21.43 g/L after 13.5 h, and the final conversion ratio of PPA was 82.38% (Wang et al., 2016). LDHs using NADH or NADPH as its co-substrate catalyze the reduction and oxidation reaction between pyruvate and lactic acid (Al-Jassabi, 2002). L-LDHs are NADH dependent, which do not have enough reduction activity if the cofactor is oxidized. However, the high cost of NADH has made it uneconomical to add pure NADH for PLA production and thus limited its application in industries (Li et al., 2019). Interestingly, glucose dehydrogenases (GDHs) can catalyze the conversion of oxygen and glucose to gluconic acid and hydrogen peroxide, while NAD^+^ is reduced to NADH, which could be used as a versatile biocatalyst for NADH regeneration. Therefore, an NADH/NAD^+^ regeneration system could be introduced into the recycle to increase the availability of NADH and improve the yield of L-PLA (Zhang et al., 2009). To break through these bottlenecks, one of the effective strategies was to excavate novel LDHs having a high activity and/or construct an efficient coenzyme NADH regeneration system.

**FIGURE. 1:**
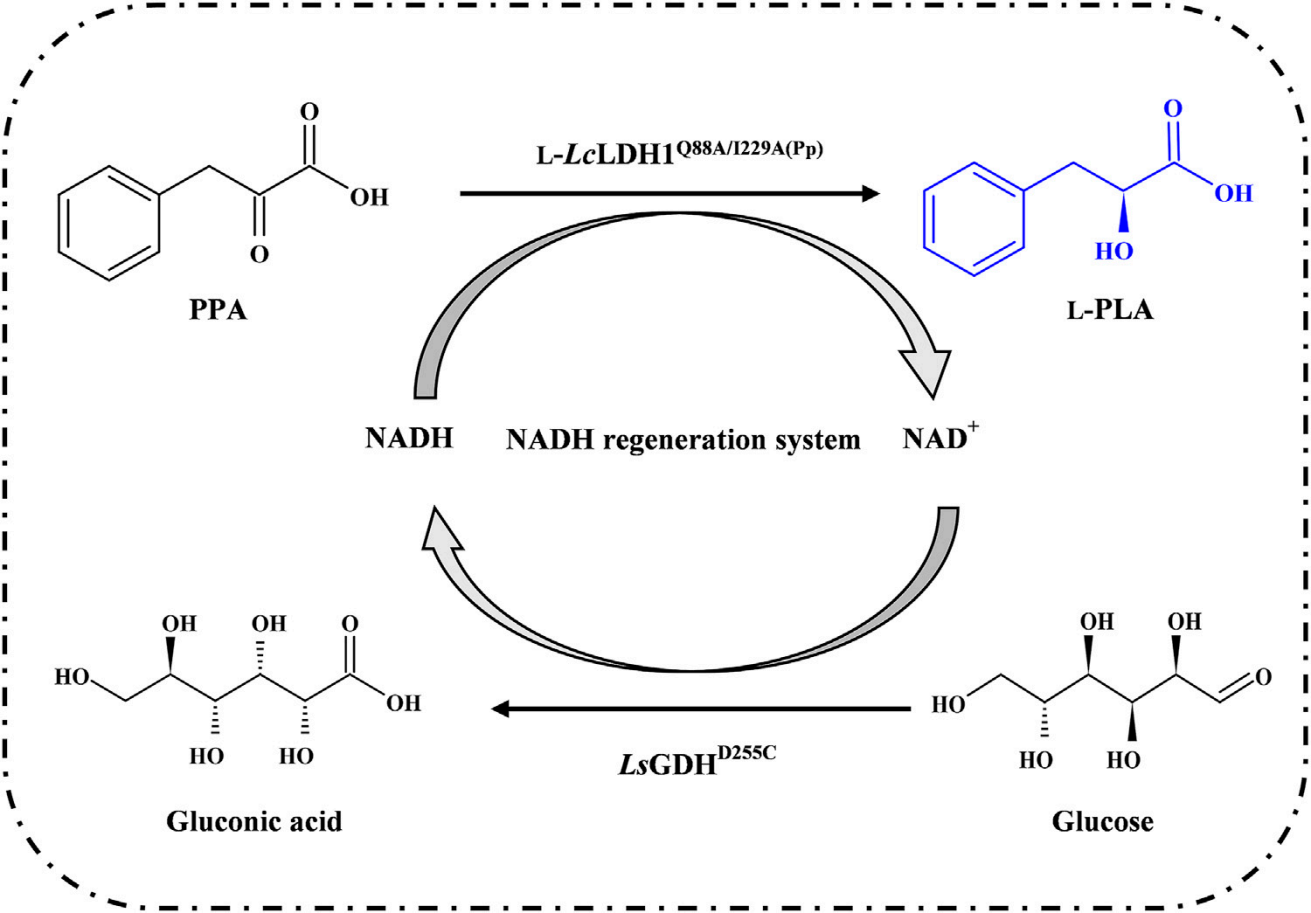
Schematic view of L-phenyllactic acid production from phenylpyruvic acid with NADH regeneration system.

Previously, two single site-directed mutagenesis of L-*Lcldh*1, L-*Lcldh*1^Q88A^, and L-*Lcldh*1^I229A^ were constructed by whole-plasmid PCR as designed theoretically and expressed in *Escherichia coli* BL21 (DE3) (Li et al., 2018). Based on homology modeling and molecular dynamics (MD) simulation, it was found that both Q88 and I229 in L-*Lc*LDH1 are located at the entrance of the substrate-binding pocket. Then, the two specific residues in L-LDH1, Gln^88^ and lle^229^, were subjected to a combinatorial double-directed mutagenesis to superimpose the superior catalytic properties of L-*Lcldh*1^Q88A^ and L-*Lcldh*1^I229A^ (Li, 2018). The catalytic performance analysis indicated that L-LDH1^Q88A/I229A^ (Ec) obtained remarkably improved specific activities towards PPA. Similar to the majority of known wild-type L-LDHs, L-LDH1^Q88A/I229A^ (Ec) displayed a lower specific activity (195.5 U/g wet cell) and catalytic efficiency (*k*_cat_/*K*_m_ = 3.7 mM^−1^ s^−1^) towards PPA, which could catalyze 10 mM PPA to L-PLA with 99% *ee*_p_ and 77% yield within 140 min. In this work, in order to improve the yield of L-PLA and avoid the formation of byproducts, L-LDH1^Q88A/I229A^ was expressed in *Pichia pastoris* GS115 successfully. Then, according to the previous findings about GDHs widely used for NADH regeneration in bioconversion process, *Ls*GDH^D250C^, *Bs*GDH^E170K/Q250L^, and SyGDH were expressed in *E. coli* BL21 (DE3), respectively. After screening, the asymmetric reduction of PPA was conducted by L-*Lc*LDH1^Q88A/I229A^ (Pp) coupling with *Ls*GDH^D250C^ (Figure 1). Finally, L-PLA was efficiently prepared by fed-batch process.

## Materials and Methods

### Materials

The strains, plasmids, and genes used in this work are summarized in Table 1. *E. coli* BL21 (DE3), *P. pastoris* GS115, pET-28a(+), and pPIC9K were used for the construction of recombinant plasmids and the expression of GDHs and L-*Lc*LDH1^Q88A/I229A^. The recombinant plasmids pET-22b-L-*Lcldh1* ^Q88A^, pET-22b-L-*Lcldh1* ^I229A^, pET-22b-L-*Lcldh1* ^Q88A/I229A^, and pET-28a-Sy*gdh* were constructed and stored in our lab. The recombinant plasmids pET-28a-*Lsgdh* ^D252C^ and pET-28a-*Bsghd* ^E170K/Q250L^ were synthesized by GENEWIZ. IPTG, PPA, NAD^+^, and L/D-PLA were purchased from Sigma-Aldrich (St. Louis, MO, United States). All the other chemical reagents used were of analytical grade and commercially available (Wuxi, China).

**TABLE 1:** Strains, plasmids, and genes used in this work.

Strains, plasmids, and genes	Resource	References
*E. coli* BL21 (DE3)	Novagen, Madison, WI	NI
*P. pastoris* GS115	Novagen, Madison, WI	NI
pET-28a(+)	Novagen, Madison, WI	NI
pPIC9K	Novagen, Madison, WI	NI
L-*Lcldh1* ^Q88A^	*Lactobacillus casei* B1192	Li et al. (2018)
L-*Lcldh1* ^I229A^	*Lactobacillus casei* B1192	Li et al. (2018)
L-*Lcldh1* ^Q88A/I229A^	*Lactobacillus casei* B1192	Li (2018)
*Sygdh*	*Thermoplasma acidophilum*	Yu et al. (2015)
*Lsgdh* ^D252C^	*Lysinibacillus sphaericus* G10	Ding et al. (2013)
*Bsghd* ^E170K/Q250L^	*Bacillus subtilis* strain 168	Vazquez-Figueroa et al. (2007)

NI, no information.

### Expression of L-LDH1^Q88A/I229A^ in *P. pastoris* GS115

L-*Lcldh*1^Q88A/I229A^ was excised from recombinant plasmid pET-22b-L-*Lcldh*1^Q88A/I229A^ by digestion with *Eco*R I and *Not* I and inserted into pPIC9K digested with the same enzymes, followed by transforming it into *E. coli* JM109. The resulting recombinant expression plasmid was then linearized with *Sal*I and transformed into *P. pastoris* GS115 by electroporation using a Gene Pulser apparatus (Bio-Rad, Hercules, CA, United States) according to the manufacturer’s instruction. All *P. pastoris* transformants were primarily screened based on their ability to grow on a MD plate and subsequently inoculated on a geneticin G418-containing YPD plate at concentration of 4.0 mg/ml for the screening of multiple copies. In total, 50 of the G418-resistant *P. pastoris* transformants were isolated and determined by colony PCR using vector-specific primers. Then, 20 *P. pastoris* transformants were selected for the flask expression test. After screening, the best transformant was induced by adding 1.0% (v/v) methanol at 24-h intervals for 72 h. Then, a total of 200 ml of cultured supernatant was harvested for further study. Comparatively, GS115*/*L-*Lcldh*1 was used as a positive control, while GS115 transformed with pPIC9K, named GS115/pPIC9K, was used as a negative control.

### Screening of GDHs

The genes *Lsgdh* ^D255C^ and *Bsghd* ^E170K/Q250L^ were synthesized by GENEWIZ (Suzhou, China) and ligated into pET-28a(+) plasmid, followed by transforming them into *E. coli* BL21 (DE3), respectively. Then, the transformants were selected on Luria–Bertani (LB) agar medium with 100 μg/ml kanamycin, and the positive clones screened and confirmed by DNA sequencing were named *E. coli*/*Lsgdh* ^D252C^ and *E. coli*/*Bsghd* ^E170K/Q250L^, respectively. *E. coli*/Sy*gdh* was constructed and stored in our lab.

### Preparation of Recombinant Proteins

The expressed recombinant L-*Lc*LDH1^Q88A/I229A^ (Pp) or L-*Lc*LDH1 (Pp) with a 6× His tag at its C-terminus was purified by affinity column chromatography. Briefly, the fermentation supernatant was centrifuged (5,000 × *g*, 10 min, 4°C) and collected. Then, the obtained supernatant (200 ml) was filtered through a 0.22-μm filter, adjusted to pH 7.8, and loaded onto a nickel-nitrilotriacetic acid column (Tiandz, Beijing, China) pre-equilibrated with binding buffer (20 mM Tris-HCl, 500 mM NaCl, and 20 mM imidazole, pH 7.8), followed by elution at a rate of 0.4 ml/min with elution buffer that was the same as the binding buffer except for 200 mM imidazole. Aliquots of 1.0 ml eluent only containing the target protein, L-*Lc*LDH1^Q88A/I229A^ (Pp) or L-*Lc*LDH1 (Pp), were pooled, dialyzed against 50 mM Na_2_HPO_4_–NaH_2_PO_4_ buffer (pH 7.0), and concentrated by ultrafiltration using a 10-kDa cutoff membrane (Millipore, Billerica, MA, United States). The target protein was assessed by SDS-PAGE, and the protein concentration was determined by the Bradford method using bovine serum albumin as the standard.

A different method of preparing GDHs was used. Firstly, *E. coli* transformant cells were cultured in LB medium supplemented with 100 μg/ml kanamycin at 37°C and 220 rpm until OD_600_ reached 0.6–0.8. After having been induced by 0.05 mM IPTG at 25°C for 10 h, *E. coli* cells were harvested by centrifugation at 4°C, resuspended in 50 mM Na_2_HPO_4_–NaH_2_PO_4_ buffer (pH 7.0), and disrupted by sonication in ice-water bath. Then, the obtained supernatant was used as cell-free extracts for crude enzyme activity assay. In order to reduce the influence of miscellaneous protein in the crude enzyme solution of the three selected GDHs, the crude enzyme solutions were treated in a hot water bath at 50°C for 1 h, and then their relative activities were measured, respectively.

### Enzyme Activity Assay

The activities of LDHs and GDHs were assayed by measuring respectively as described previously (Zhu et al., 2017; Li et al., 2018), with a slight modification. L-LDH activity on PPA was assayed by the reaction mixture containing 50 mM sodium acetate–acetate buffer (pH 5.0), 2 mM NADH, and 10 mM PPA. One activity unit (U) of LDH activity was defined as the amount of enzyme catalyzing the reduction of 1 µmol PPA per minute under the given assay conditions (40°C, 5 min). GDH activity was measured by the reaction mixture containing 50 mM sodium acetate–acetate buffer (pH 5.0), 2 mM NAD+, and 40 mM glucose. One activity unit (U) of GDH was defined as the amount of enzyme that produced 1 µmol NADH per minute under the given assay conditions (40°C, 5 min).

### Determination of NAD^+^ and *Ls*GDH^D255C^ on L-PLA production

The recombinant *E. coli*/*Lsgdh* ^D252C^ cells were induced, harvested, and disrupted by sonication; then, the obtained supernatant was incubated for 1 h at 50°C. To determine the effect of the addition of NAD^+^ on L-PLA production, a bioconversion reaction was performed in the final volume of 5 ml of 50 mM sodium acetate–acetate buffer system (pH 5.0) containing 100 mM PPA, 120 mM glucose, 10.0 U/ml L-*Lc*LDH1^Q88A/I229A^ (Pp), 5.0 U/ml *Ls*GDH^D250C^, and NAD^+^ at elevated concentrations ranging from 0.025 to 0.50 mM, respectively, at 40°C and 200 rpm for 1 h. Furthermore, using L-PLA production as the criteria, bioconversion reactions, in the final volume of 5 ml of 50 mM sodium acetate–acetate buffer system (pH 5.0) containing 100 mM PPA, 120 mM glucose, 10.0 U/ml L-*Lc*LDH1^Q88A/I229A^ (Pp), 0.1 mM NAD^+^, and *Ls*GDH^D250C^ at the concentration range of 1.0–6.0 U/ml, were conducted, respectively, at 40°C and 200 rpm for 1 h. After completion of the reactions, the samples were treated in boiling water bath at 100°C for 10 min and centrifuged at 12,000 rpm for 5 min. The concentrations of PPA and L-PLA in the resulting supernatants were quantitatively analyzed by high-performance liquid chromatography (HPLC).

### Bioconversion of PPA to L-PLA by Coupling L-*Lc*LDH1^Q88A/I229A^ (Pp) With *Ls*GDH^D250C^

The bioconversion was initiated in 50 mM sodium acetate–acetate buffer (pH 5.0) containing 100 mM PPA, 120 mM glucose, 0.1 mM NAD^+^, 10.0 U/ml L-*Lc*LDH1^Q88A/I229A^ (Pp), and 4.0 U/ml *Ls*GDH^D250C^ at 40°C. Furthermore, a higher substrate concentration (200 mM PPA) was used to verify substrate inhibition. Then, fed-batch bioconversion was performed in a 250-ml beaker containing 100 ml of the reaction mixture. The initial reaction mixtures containing 100 mM PPA, 120 mM glucose, 0.1 mM NAD^+^, 10.0 U/ml L-*Lc*LDH1^Q88A/I229A^ (Pp), and 4.0 U/ml *Ls*GDH^D250C^ were incubated at 40°C and 200 rpm. PPA powder (4.93 g) and glucose powder (6.48 g) were both averagely supplemented at 0.5, 1, and 2 h, respectively. During the bioconversion process, aliquots of 500-μl samples were drawn out periodically and then analyzed by HPLC.

### Analytical Methods

PPA and PLA were assayed by inversed-HPLC using a Waters e2695 apparatus (Waters, Milford, MA, United States) equipped with a Prontosil C18 AQ chromatographic column. The mobile phase of methanol/H_2_O (4:6, v/v) with 0.05% acetocaustin (v/v) was used at a flow rate of 0.8 ml/min and monitored using a 2489 UV–Vis detector at 210 nm. The yield of L-PLA (c_m_) was calculated using the equation: *c*_m_ = (*c*_p_ / *c*_s_) × 100%, in which *c*_p_ was the concentration of PLA (mM), and *c*_s_ was the concentration of PPA (mM).

Different from that of inversed-HPLC, positive-phase HPLC equipped with an OD-H chiral column (Daicel, Tokyo, Japan; 4.6 mm × 250 mm, 5 μm) was used to analyze the optical purity of L-PLA under the same assay conditions as described above, except for the mobile-phase n-hexane/isopropanol (98:2, v/v) with 0.05% acetocaustin (v/v). The enantiomeric excess product (*ee*_p_) of L-PLA was calculated by using the equation: *ee*_p_ = [(*A*_L_ - *A*_D_) / (*A*_L_ + *A*_D_)] × 100%, where *A*_L_ and *A*_D_ are the concentrations of L-PLA and D-PLA, respectively. The space time yield (STY) and the average turnover frequency (aTOF) were calculated by using the equations: STY (g/L/h) = *C*_p_ / *t*, aTOF (g/g/h) = *C*_p_ / (*t* × *C*_e_), in which *C*_p_ was the concentration of L-PLA (g/L), *t* was the reaction time, and *C*_e_ was L-*Lc*LDH1^Q88A/I229A^ (Pp) protein concentration (g/L).

## Results and Discussion

### Enzyme Assay of L-*Lc*LDH1^Q88A/I229A^ (Pp) Expressed in *P. pastoris* GS115

**FIGURE. 2:**
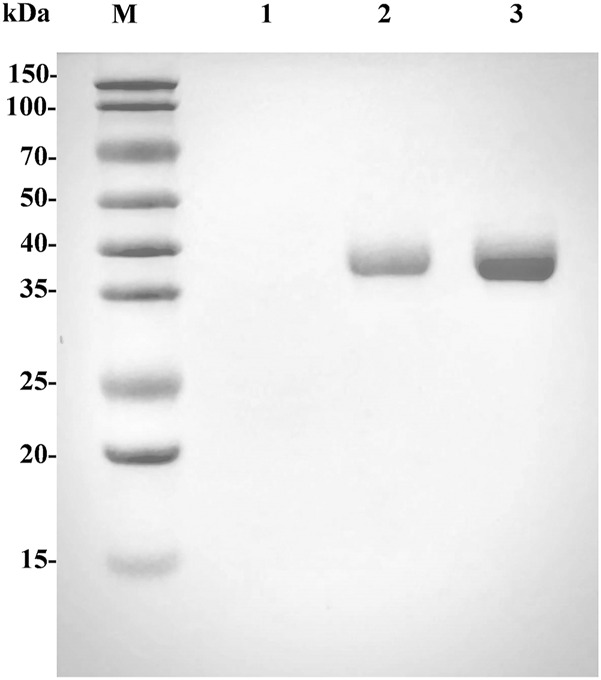
SDS-PAGE analysis of the expressed and purified L-*Lc*LDH1^Q88A/I229A^ (Pp). Lane M, standard protein molecular mass markers; lane 1, the cultured supernatant of GS115/pPIC9K; lane 2, the cultured supernatant of GS115/L-*lcldh1* ^Q88A/I229A^; lane 3, the purified L-*Lc*LDH1^Q88A/I229A^ (Pp).

To improve the L-phenyllactic acid (L-PLA) production efficiency and reduce costs, we attempted the expression of *L-LcLDH1* ^Q88A/I229A^ in *P. pastoris* GS115. The fermentation supernatant and the purified L-*Lc*LDH1^Q88A/I229A^ (Pp) displayed one single protein band with an apparent molecular weight of approximately 36.8 kDa on SDS-PAGE gels (Figure 2, lanes 2 and 3), equal to the theoretical one (36.75 kDa) of L-*Lc*LDH1^Q88A/I229A^, which indicated that the L-*Lc*LDH1^Q88A/I229A^ (Pp) was expressed successfully in *P. pastoris* GS115. The specific activity of L-*Lc*LDH1^Q88A/I229A^ (Pp) was as high as 447.6 U/mg towards PPA at the optimum temperature and pH of 40°C and 5.0, which was obviously higher than the other L-LDHs reported by Zhu (28.11 U/g) (Zhu et al., 2017), Jia (71.06 U/g) (Jia et al., 2010), Aslan (51.3 U/g) (Aslan et al., 2016), and Zheng (72.6 U/g) (Zheng et al., 2011). Many studies regarding engineering of target enzymes have been reported, while almost all research demonstrated the feasibility of improving the L-LDH activity towards PPA by site-directed mutagenesis. As a key enzyme in L-PLA production, most of the L-LDHs reported were expressed in *E. coli*, which exhibited low catalytic efficiency (*k*_cat_/*K*_m_), production efficiency, and yield and produced many more byproducts. Here L-*Lc*LDH1^Q88A/I229A^ (Pp) expressed in *P. pastoris* GS115 was different from all the previously reported LDHs, which had a higher enzyme activity and catalytic efficiency. Moreover, one of the advantages of the *P. pastoris* expression system was that the purities of the expressed recombinant proteins were very high, which could greatly simplify the purification processes. *P. pastoris* enables some post-translational modifications of a recombinant protein, such as glycosylation, whereas there is no N-glycosylation site in this work. The difference of L-*Lc*LDH1^Q88A/I229A^ expressed in the two expression systems will be further investigated in the following work.

**FIGURE. 3:**
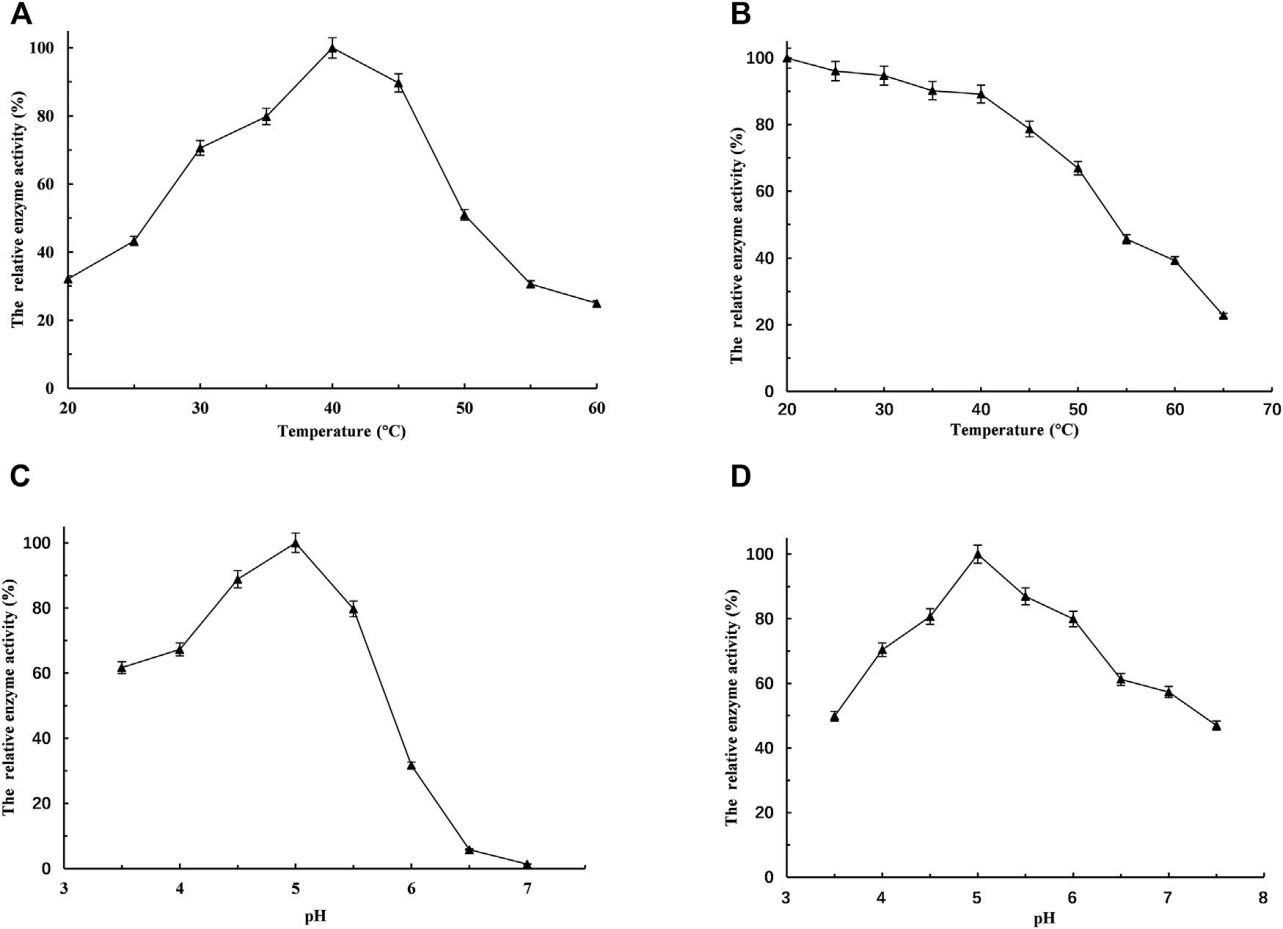
Optimization of bioconversion conditions of the recombinant L-*Lc*LDH1^Q88A/I229A^ (Pp). **(A)** The temperature optima were measured under the standard assay conditions, but temperatures ranged from 20 to 60°C. **(B)** To estimate the temperature stability, the recombinant L-*Lc*LDH1^Q88A/I229A^ (Pp) was incubated for 2 h from 20 to 65°C, respectively. **(C)** The pH optima were measured under the above-mentioned assay conditions, except for pH ranging from 3.5 to 7.0. **(D)** To estimate the pH stability, the recombinant L-*Lc*LDH1^Q88A/I229A^ (Pp) was incubated for 1 h in a different pH buffer at 4°C, and then the relative activity was determined at the optimal temperature of 40°C and optimal pH of 5.0. Means and standard deviations of triplicate experiments are shown.

To determine the influence of temperature on the activity of L-*Lc*LDH1^Q88A/I229A^ (Pp), the temperature–activity profiles were assessed from 20 to 60°C with PPA as the substrate (Figure 3A). To evaluate the temperature stabilities, the residual activities were measured after incubation at various temperatures for 2.0 h (Figure 3B). The optimum temperature was at 40°C (Figure 3A), and L-*Lc*LDH1^Q88A/I229A^ (Pp) was stable at temperatures below 45°C (Figure 3B). Within the range of the thermal tolerance of L-*Lc*LDH1^Q88A/I229A^ (Pp), the high temperature increased the solubility and dissolution rate of PPA, contributing to the effective conversion of PPA into L-PLA at a high concentration and with a high yield. Because pH would considerably affect the enzyme activity and stability in application, the influence of pH on L-*Lc*LDH1^Q88A/I229A^ (Pp) was also assessed. Its optimal pH was at 5.0 (Figure 3C), and it was stable at 4.5–5.0 (Figure 3D). Then, the half-life value was measured at its optimal temperature and pH (40°C, 5.0). During the process, aliquots of 50-μl enzyme samples were drawn out, and their relative enzyme activities were determined. The half-life value calculated was 6.1 h, which was longer than that of L-*Lc*LDH1^Q88A/I229A^ (Ec) (4.4 h) (data not shown). Next, taking economic efficiency and catalytic activity into consideration, L-*Lc*LDH1^Q88A/I229A^ (Pp) expressed in *P. pastoris* exhibited better superiority than L-*Lc*LDH1^Q88A/I229A^ (Ec) expressed in *E. coli*.

The kinetic parameters of the recombinant enzymes were determined using PPA as substrate (Table 2). The *K*_m_ of L-*Lc*LDH1^Q88A/I229A^ (Pp) towards PPA was 4.08 mM, which was 57.1% lower than that of wild-type enzyme L-*Lc*LDH1. Moreover, the *k*_cat_/*K*_m_ of L-*Lc*LDH1^Q88A/I229A^ (Pp) (94.3 mM^−1^ s^−1^) was significantly increased, which was 67.4 times higher than that of L-*Lc*LDH1 (Pp) (1.4 mM^−1^ s^−1^). This study also found that the *V*_max_ (627.0 U/mg) and the catalytic efficiency *k*_cat_/*K*_m_ of L-*Lc*LDH1^Q88A/I229A^ (Pp) were 26.1 times and 25.5 times higher, respectively, than those of L-*Lc*LDH1^Q88A/I229A^ (Ec) (24.0 U/mg and 3.7 mM^−1^ s^−1^) expressed in *E. coli.* Therefore, for bioconversion of PPA to L-PLA, an appropriate L-LDH with a high PPA specificity and specific activity is required. The L-*Lc*LDH1^Q88A/I229A^ (Pp) in this study is a relatively good candidate.

**TABLE 2:** The kinetic parameters of the recombinant enzymes.

Recombinant enzymes	*K*_m_ (mM)	*V*_max_ (U/mg)	*k*_cat_ (s^−1^)	*k*_cat_/*K*_m_ (mM^−1^·s^−1^)
L-*Lc*LDH1^Q88A/I229A^ (Pp)	4.08 ± 0.12	627.0 ± 17	385 ± 12	94.3
L-*Lc*LDH1 (Pp)	9.52 ± 0.26	22.5 ± 0.58	13.8 ± 0.28	1.4
L-*Lc*LDH1^Q88A/I229A^ (Ec)	3.84 ± 0.11	24.0 ± 0.45	14.7 ± 0.14	3.7

L-*Lc*LDH1^Q88A/I229A^ (Pp), double-site mutant enzyme of L-*Lc*LDH1 expressed in *P. pastoris*; L-*Lc*LDH1 (Pp), the wild-type enzyme expressed in *P. pastoris*; L-*Lc*LDH1^Q88A/I229A^ (Ec), double-site mutant enzyme of L-*Lc*LDH1 expressed in *E. coli*.

### Screening of Coenzymes Based on the Preference for NAD^+^

**FIGURE. 4:**
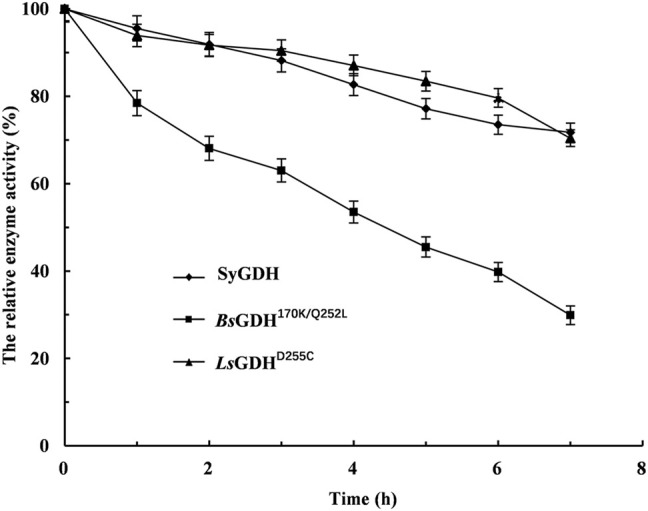
Effect of the time of heat treatment on the stability of glucose dehydrogenases under the optimal catalytic conditions of 40°C and pH 5.0. ◆, the cell lysate of induced *E. coli/*Sy*gdh*; ■, the cell lysate of induced *E. coli/Bsgdh* ^170k/Q252L^; ▲, the cell lysate of induced *E. coli/Lsgdh* ^D255C^. Means and standard deviations of triplicate experiments are shown.

After *E. coli* cells sonication, the GDH activities were measured at 50°C, respectively. Then, after incubation for 1 h at 50°C, their relative activities were measured. The *Ls*GDH^D255C^ showed the highest specific activity (11.1 ± 0.21 U/mg) and still retained 97.3% of its initial activity after incubation for 1 h at 50°C without any protective agent (Table 3). Because L-*Lc*LDH1^Q88A/I229A^ (Pp) is acidophilic and sensitive to pH, the pH of the reaction mixture should be consistent with L-*Lc*LDH1^Q88A/I229A^ (Pp). Before coupling with L-*Lc*LDH1^Q88A/I229A^ (Pp), it was necessary to determine the stability of the three GDHs under optimal catalytic conditions (40°C, pH 5.0). Under the conditions of 40°C and pH 5.0, the relative enzyme activities of GDHs are shown in Figure 4. The relative enzyme activity of GDHs was significantly decreased with increasing reaction time, while that of SyGDH and *Ls*GDH^D255C^ was decreased at a slower reaction rate. Subsequently, the half-life values of GDHs were measured at 40°C and pH 5.0. The results showed that the half-life value of *Bs*GDH^170k/Q252L^ was 4.5 h, which was the lowest of the three GDHs and shorter than that of L-*Lc*LDH1^Q88A/I229A^ (Pp) (6.1 h). The half-life value of *Ls*GDH^D255C^ was 18.5 h, longer than that of SyGDH (13.8 h). Combined with the latest research results of our team on SyGDH, the *K*_m_ and catalytic efficiency (*k*_cat_ */K*_m_) of SyGDH towards NADP^+^ were 0.67 mM and 104.0 mM^−1^s^−1^, respectively, while they were 157.9 mM and 0.64 mM^**−**1^s^**−**1^ towards NAD^+^, suggesting that it preferred NADP^+^ as a coenzyme rather than NAD^+^ (Hu et al., 2020). Based on the above-mentioned study, *Ls*GDH^D255C^ with its good affinity for NAD^+^, high thermal stability, and acid resistance was the best choice as a coenzyme for coupling with L-*Lc*LDH1^Q88A/I229A^ (Pp) to be introduced into the coenzyme regeneration system.

**TABLE 3:** Analysis of the activities of the three glucose dehydrogenases before and after heat treatment.

Item	SyGDH	*Bs*GDH^E170K/Q252L^	*Ls*GDH^D255C^
Before (U/mg)	0.865 ± 0.025	4.99 ± 0.17	11.1 ± 0.21
After (U/mg)	0.806 ± 0.022	4.72 ± 0.11	10.8 ± 0.29
Relative activity (%)	93.2 ± 0.88	94.6 ± 0.65	97.3 ± 1.38

### Optimization of Bioconversion Conditions

**FIGURE. 5:**
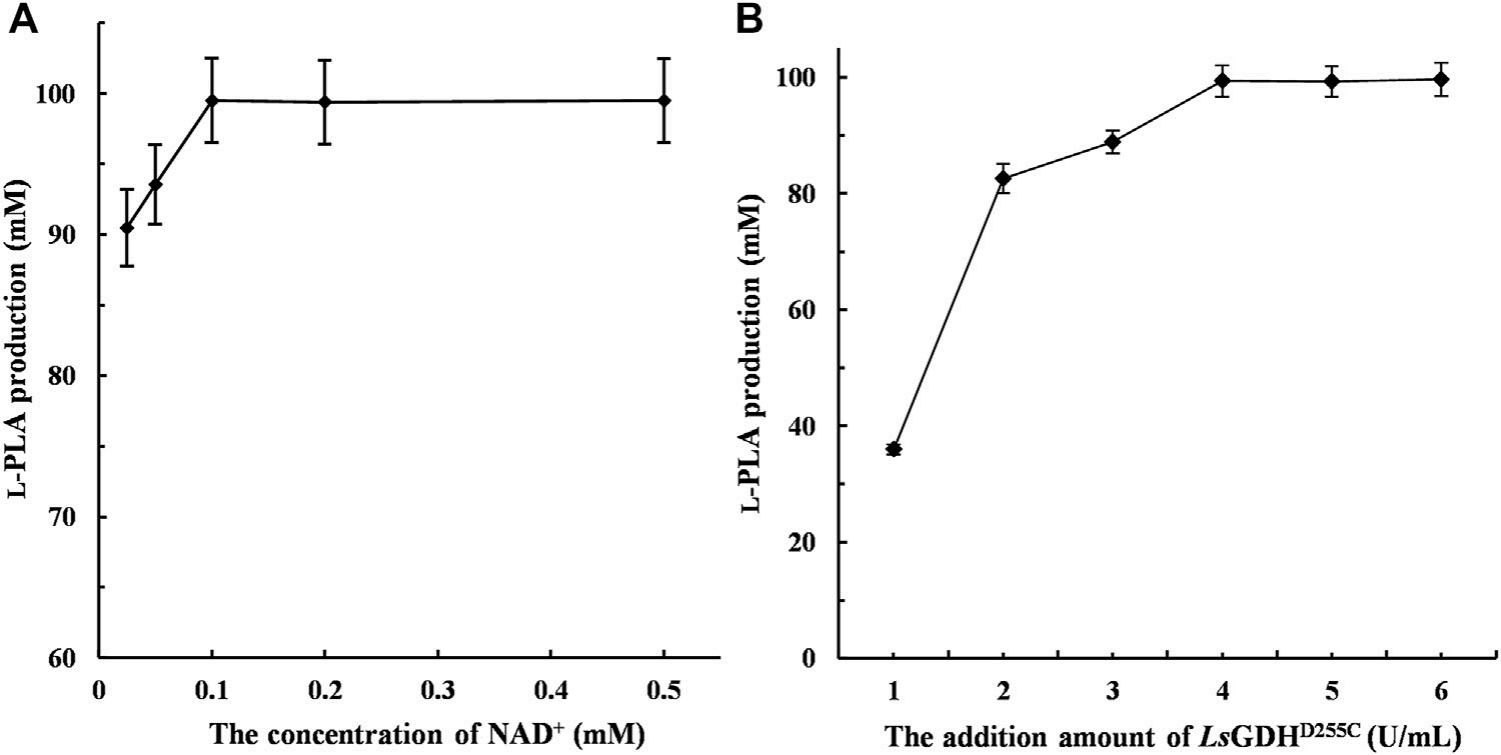
Effect of the concentration of NAD^+^ and *Ls*GDH^D255C^ on L-phenyllactic acid (L-PLA) production. **(A)** Effect of the concentration of NAD^+^ on L-PLA production. **(B)** Effect of the *Ls*GDH^D255C^ amount on L-PLA production. The bioconversion was conducted in 50 mM sodium acetate–acetate buffer (pH 5.0) containing 100 mM PPA and 120 mM glucose at 40°C for 1 h. Means and standard deviations of triplicate experiments are shown.

The large-scale production of L-PLA from PPA requires large amounts of NADH and therefore has limited applications in the industry. In this study, NADH/NAD^+^ can be transformed reversibly by coupling L-*Lc*LDH1^Q88A/I229A^ (Pp) with *Ls*GDH^D225C^, thereby maintaining the intracellular redox balance. The initial activity of the reaction mixture was an important factor that affected L-PLA production. To investigate the effect of NAD^+^ on L-PLA production, the concentrations of NAD^+^ from 0.025 to 0.5 mM were determined as shown in Figure 5A. High L-PLA production was achieved in a broad range of NAD^+^ from 0.025 to 0.5 mM, while the highest L-PLA production could be obtained at a low concentration of 0.1 mM.

Compared with a single-enzyme [L-LDH1^Q88A/I229A^ (Pp)] reaction, no or little byproducts were generated in this coenzyme regeneration system (Supplementary Figure S1). Moreover, *Ls*GDH^D225C^ can be utilized to regenerate NADH because of its low-cost substrate (glucose). Therefore, the effect of adding an amount of *Ls*GDH^D225C^ on L-PLA production was determined, and it was shown that L-PLA production was significantly improved with increasing enzyme amounts of *Ls*GDH^D225C^ up to 6.0 U/ml and that the highest L-PLA production (>99.9 mM) with over 99% yield could be obtained at a low concentration of 4.0 U/ml (Figure 5B).

**FIGURE. 6:**
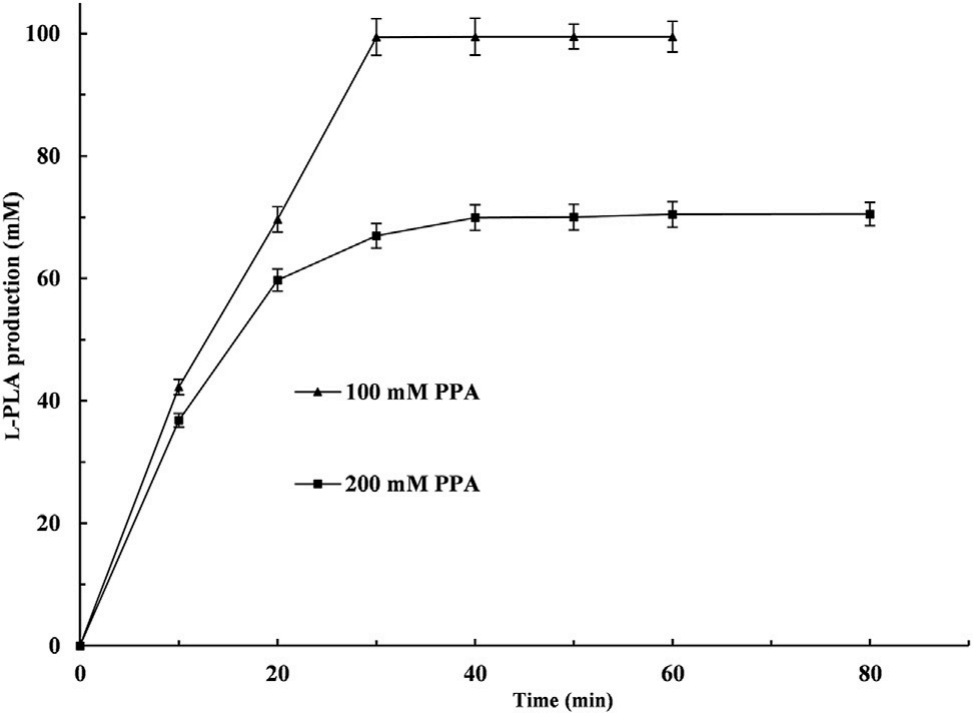
Time course of L-phenyllactic acid production from phenylpyruvic acid (PPA) by coupling L-*Lc*LDH1^Q88A/I229A^ (Pp) with *Ls*GDH ^D225C^ *in vitro*. ▲, PPA concentration of 100 mM; ■, PPA concentration of 200 mM. The bioconversion was conducted in 50 mM sodium acetate–acetate buffer (pH 5.0) containing 100 or 200 mM PPA and 120 mM glucose at 40°C within 80 min. Means and standard deviations of triplicate experiments are shown.

Under the above-mentioned optimal conditions, 100 and 200 mM PPA were used to investigate the potential for the production of L-PLA, respectively. As shown in Figure 6, 100 mM PPA could be almost completely hydrolyzed (99.4% yield) at 50°C within 30 min. When the PPA concentration was up to 200 mM and the reaction time was prolonged to 80 min, the yield of L-PLA was only 35.0%. Moreover, 200 mM PPA showed obvious inhibitory effects on *Ls*GDH^D255^ or/and L-LDH1^Q88A/I229A^ (Pp) in the biotransformation process. Similarly, substrate inhibition is a universal problem in an enzymatic reaction (Li et al., 2008; Lin et al., 2008; Reed et al., 2010), which can be alleviated by intermittent feeding with PPA to produce a high amount of L-PLA.

### Large-Scale Production by an Enzyme Coupling System by Fed-Batch Biotransformation

**FIGURE. 7:**
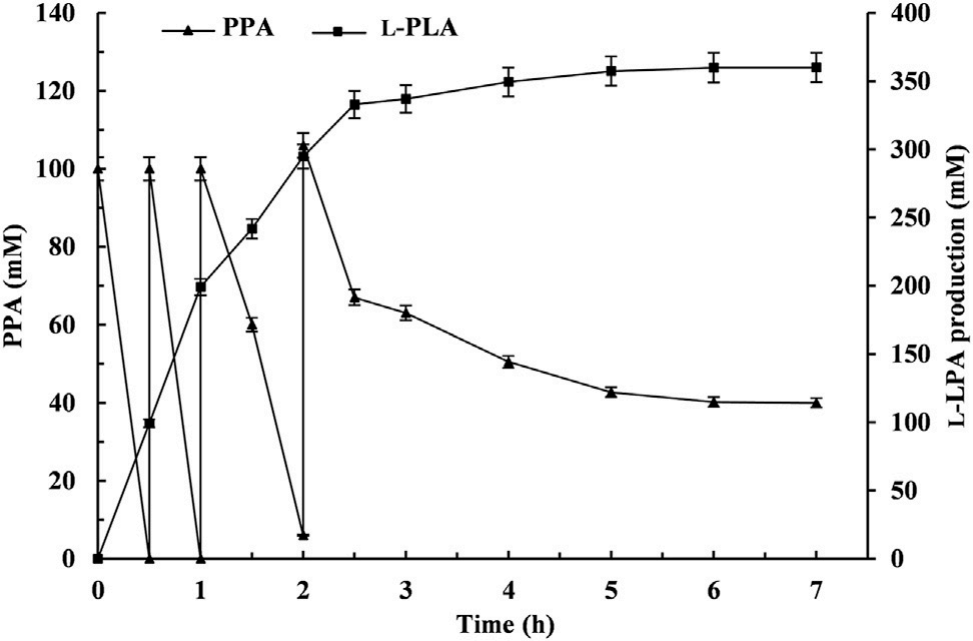
Time course of the fed-batch production of L-phenyllactic acid (L-PLA) by coupling L-*Lc*LDH1^Q88A/I229A^ (Pp) with *Ls*GDH ^D225C^ *in vitro*. ▲, phenylpyruvic acid concentration; ■, L-PLA production. Means and standard deviations of triplicate experiments are shown.

The fed-batch bioconversion was carried out with 100 ml 50 mM sodium acetate–acetate buffer system (pH 5.0) containing initial PPA and glucose concentrations of 100 and 120 mM under the abovementioned optimal conditions. L-PLA was rapidly accumulated with the decrease of PPA during the first 0.5 h, and then PPA powder (4.92 g) and glucose powder (6.48 g) feedings were performed at 0.5, 1, and 2 h, respectively. Subsequently, it went on until 7 h without further additions. As shown in Figure 7, the reaction can be divided into two stages: rapid reaction stage (0–2.5 h) and slow reaction stage (2.5–6 h). With the feeding of the substrates PPA and glucose during the fed-batch conversion process, the L-PLA concentration was continuously increasing at a relatively high rate before the third feeding. After incubation for 6 h, 400 mM PPA was almost completely hydrolyzed, affording L-PLA with 99.9% *ee*_p_, 90% yield (359.8 mM), and a high STY of 10 g/L/h and aTOF of 269.3 g/g/h. Additionally, the number of coenzyme cycles was 3,598, which was 3.6 times than that of 100 mM PPA (995).

Several biosynthesis methods, mostly by means of whole cells, have been reported for L-PLA production. However, highly stereoselective and efficient biosynthesis of L-PLA was rare—for example, a NADH regeneration system developed in *Lactobacillus plantarum* by expressing the *fdh* gene coding for formate dehydrogenase from *Candida boidinii* was used to convert PPA to L-PLA, but only 85.24 mM L-PLA with a low yield of 71.03% and STY of 0.9 g/L/h was produced (Li et al., 2019). Recently, 103.8 mM L-PLA (*ee*_p_ > 99.7%) was produced by using whole cells (OD_600_ = 25) of recombinant *E. coli/*Duet-*ldh*L-*gdh* co-expressing L-lactate dehydrogenase from *L. plantarum* and GDH from *Bacillus megaterium*, but the general conversion ratio of 55.8% was still unsatisfactory (Zhu et al., 2017). In our study, 359.8 mM L-PLA with 99.9% *ee*_p_ was achieved from 400 mM PPA, at an excellent conversion ratio of 90%, which were 3.47 and 1.61 times higher than that of *E. coli* reported by Zhu, respectively (Zhu et al., 2017). The above-mentioned results indicated that the cofactor-dependent biotransformation system was imperative for high productivity and conversion ratio. Moreover, the optically pure L-PLA and high conversion ratio of PPA could be reached by a fed-batch bioconversion mode in an enzyme coupling system *in vitro*.

## Conclusion

In conclusion, a NADH regeneration system was introduced into L-PLA production. Firstly, L-LDH1^Q88A/I229A^ (Pp) was expressed in *P. pastoris* GS115 successfully. Additionally, a glucose dehydrogenase variant *Ls*GDH^D255C^ with good affinity for NAD^+^ was screened and successfully introduced into the coenzyme NADH regeneration system. This NADH regeneration system obviously increased the space–time yield and total turnover number of coenzyme NAD^+^. Benefiting from coenzyme regeneration, the accumulation of intermediate Phe dramatically decreased, and L-PLA production dramatically increased in fed-batch bioconversion. The coenzyme NADH regeneration system can be considered a promising strategy for increasing the yield of highly optically pure L-PLA at high productivity and high stereoselectivity.

## Data Availability

The original contributions presented in the study are included in the article/Supplementary Material, further inquiries can be directed to the corresponding author.
