# Supplementary material for: Enantioselective Biosynthesis of L-Phenyllactic Acid From Phenylpyruvic Acid In Vitro by L-Lactate Dehydrogenase Coupling With Glucose Dehydrogenase
Source: Front Bioeng Biotechnol. 2022 Feb 18;10:846489. doi: 10.3389/fbioe.2022.846489 (PMC8894805; doi:10.3389/fbioe.2022.846489)
Supplement: Supplementary file 1 [file Image1.pdf]

Supplementary Information

Enantioselective biosynthesis of L-phenyllactic acid by coupling L-lactate dehydrogenase with glucose dehydrogenase

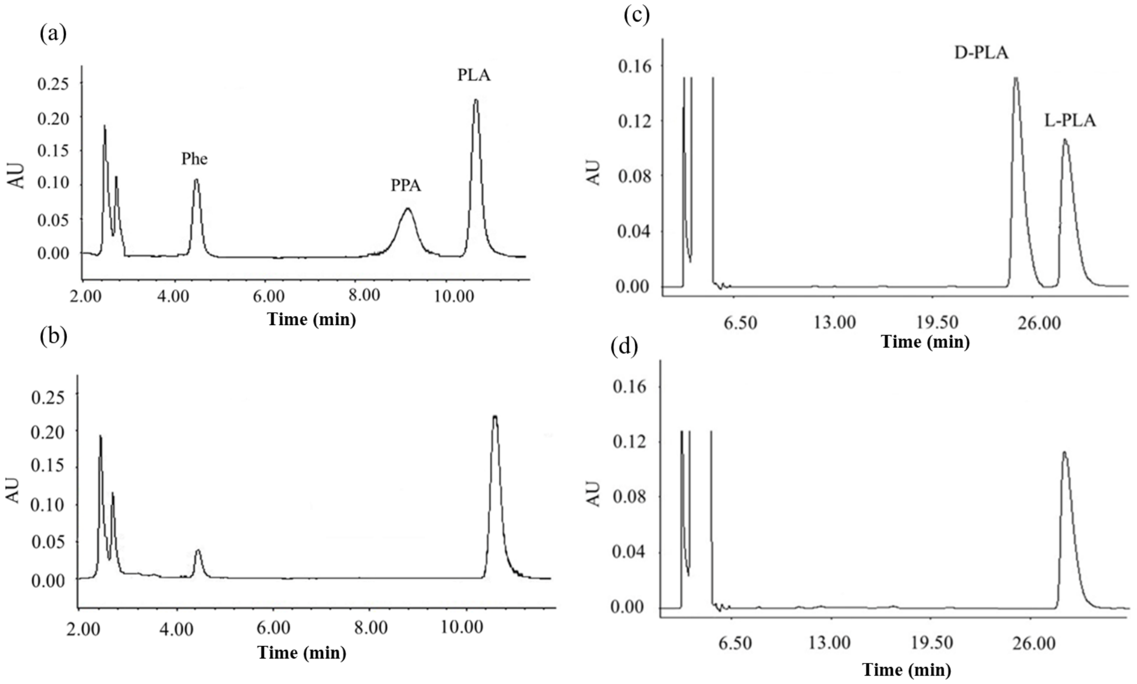

Fig. S1. HPLC analysis of the asymmetric reduction of PPA catalyzed by L-*Lc*LDH1$^{Q88A/I229A}$(Pp) and L-*Lc*LDH1$^{Q88A/I229A}$(Pp) coupling with *Ls*GDH^{D225C}, respectively. (a) Standard samples of PPA、 Phe、 PLA. (b) The product analysis PLA catalyzed PPA by L-*Lc*LDH1$^{Q88A/I229A}$(Pp). (c) Standard samples of L/D-PLA. (d) The product analysis L-PLA catalyzed PPA by L-*Lc*LDH1$^{Q88A/I229A}$(Pp) coupling with *Ls*GDH^{D225C}.
